# Characterization of the interaction between β-catenin and sorting nexin 27: contribution of the type I PDZ-binding motif to Wnt signaling

**DOI:** 10.1042/BSR20191692

**Published:** 2019-11-13

**Authors:** Brian J. DuChez, Christina L. Hueschen, Seth P. Zimmerman, Yvonne Baumer, Stephen Wincovitch, Martin P. Playford

**Affiliations:** 1 Cell Biology and Physiology Center, National, Heart, Lung, and Blood Institute, National Institutes of Health, Bethesda, MD 20982, U.S.A.; 2 Cytogenetics and Microscopy Core, National Human Genome Research Institute, National Institutes of Health, Bethesda, MD 20892, U.S.A.

**Keywords:** beta-catenin, endosome, sorting nexin

## Abstract

Background: Sorting Nexin 27 (SNX27) is a 62-kDa protein localized to early endosomes and known to regulate the intracellular trafficking of ion channels and receptors. In addition to a PX domain common among all of the sorting nexin family, SNX27 is the only sorting family member that contains a PDZ domain. To identify novel SNX27–PDZ binding partners, we performed a proteomic screen in mouse principal kidney cortical collecting duct cells (mpkCCD) using a GST-SNX27 fusion construct as bait. We found that the C-terminal type I PDZ binding motif (DTDL) of β-catenin, an adherens junction scaffolding protein and transcriptional co-activator, interacts directly with SNX27. Using biochemical and immunofluorescent techniques, β-catenin was identified in endosomal compartments where co-localization with SNX27 was observed. Furthermore, E-cadherin, but not Axin, GSK3 or Lef-1 was located in SNX27 protein complexes. While overexpression of wild-type β-catenin protein increased TCF-LEF dependent transcriptional activity, an enhanced transcriptional activity was not observed in cells expressing β-Catenin ΔFDTDL or diminished SNX27 expression.

These results imply importance of the C-terminal PDZ binding motif for the transcriptional activity of β-catenin and propose that SNX27 might be involved in the assembly of β-catenin complexes in the endosome.

## Introduction

Many signaling pathways are regulated by endocytosis. This process plays a critical role in signaling component activation, transduction of downstream signals and signal termination. The endosomal network is composed of several intracellular compartments that begin at the plasma membrane where cargo such as a ligand-bound receptor or ion channel is internalized into the early sorting endosome [1–3]. From here, cargo sorting is initiated leading to either plasma membrane recycling or endolysosomal degradation [1–3]. Regulation of cargo fate has profound effects on downstream signaling output.

The sorting nexin family of proteins are involved in endocytosis and endosomal trafficking [4–7]. Each sorting nexin (SNX) contains a phox homology (PX) domain that binds phosphoinositide-enriched membranes [4,5,7]. To date, there are 33 identified mammalian sorting nexins that can be subdivided into three groups based on structural features [5,6]. SNX^PX-only^ proteins consist of a lone PX domain with flanking regions of non-conserved sequences. SNX^PX-BAR^ proteins contain BAR (Bin/Amphiphysin/Rvs) domains that are capable of detecting and inducing membrane curvature [6]. SNX^PX-other^ proteins contain diverse domain architectures consisting of SH3, FERM or RGS (regulators of G-protein signaling) domains among others [4,5,7].

In the latter category, sorting nexin 27 (SNX27) contains a unique Postsynaptic density protein-95, Discs-large, Zona-occludens-1 (PDZ) domain and a FERM-like/Ras-Association domain [5]. The PDZ domain is a common protein–protein interaction domain, which typically binds short peptide motifs found on the C-termini of interacting proteins [8,9].

Various studies have demonstrated that many of the SNX27-binding proteins, which include transmembrane receptors, ion channels and scaffolding proteins, conform to the consensus type-1 PDZ-binding motif D/E-S/T-X-L/V, where ‘X’ is any amino acid [10–15]. In a comprehensive proteomic sweep of cell surface proteins, Steinberg et al*.* found over 80 cell surface transmembranous proteins interact with SNX27 [15]. These proteins included glucose, zinc and amino acid transporters as well as numerous signaling receptors [15].

As each novel SNX27-interacting protein is identified our knowledge of the function of SNX27 that is becoming more apparent. For example, Lunn et al. have proposed that SNX27 regulates Kir3 potassium channel endocytosis and lysosomal degradation while data by Lauffer et al*.* suggest a role for SNX27 in the recycling of β2-adrenoreceptors from the early endosome to the plasma membrane [13,14]. Here, SNX27 functions as an adaptor protein linking this receptor to the retromer-mediated transport system via association with the Wiskott–Aldrich Syndrome Protein and SCAR Homolog (WASH) complex [16].

Previous work from our lab has expanded a role for SNX27 as a scaffold in the trafficking of intracellular proteins such as β-Pix/Git and ZO-2, in the regulation of cell motility and paracellular transport in epithelial cells [11,17]. Here, we add β-catenin to the list of SNX27-PDZ interacting proteins. β-Catenin serves as both a transcriptional end point of a growth factor (Wnt) signal and as link between cadherin proteins and the actin cytoskeleton at sites of cell–cell contact [18].

The Wnt signaling pathway is highly conserved across species and regulates many normal and pathogenic cellular processes such as embryonic development and cancer [19]. The importance of Wnt in these multiple processes has prompted numerous investigations into the Wnt-signaling pathway [20]. The pathway is initiated by interaction of numerous Wnt ligands to the Frizzled family receptors, which activates a canonical signaling pathway leading to the stabilization of β-catenin and increased transcription of target genes [20,21]. Wnt can also activate a non-canonical pathway to stimulate cytoskeletal organization or calcium mobilization [20,22].

In addition to a role in Wnt signaling, β-catenin has a well-characterized role at the adherens junction where it interacts with the cytoplasmic portion of cadherin proteins enabling homotypic adhesion between adjacent cells [23]. Simultaneously, β-catenin binds α-catenin leading to the interaction to actin and actin-binding proteins [23]. Hence, changes in cell–cell contacts may transduce signals that lead to altered actin organization or gene expression [23]. During conditions of junction remodeling, internalized cadherin proteins have been identified in the endosome, in a clathrin-dependent manner (reviewed in [23,24]). The interaction of cadherin with β-catenin has been shown to be important for exit of cadherin from vesicular structures including the early endosome [25]. However, the mechanism by which β-catenin and/or cadherin proteins are retained in endosomal sites is unclear.

As expected, the interaction of β-catenin with SNX27 is dependent on the C-terminal type-I PDZ binding motif (DTDL) of β-catenin. In the present study, we have examined the role of this novel protein–protein interaction on recruitment of β-catenin to the early endosome, to sites of cell–cell contact and transcriptional activity.

## Methods

### Antibodies

A polyclonal antibody to SNX27 was generated as described previously [11]. A monoclonal antibody against fusion proteins tagged with c-Myc (clone 9E10) and polyclonal giantin antibody (# ab93281) were purchased from Abcam (Cambridge, MA). β-Catenin, E-cadherin, GSK3, EEA1 and plakoglobin monoclonal antibodies were purchased from BD Biosciences (Franklin Lakes, New Jersey). The Cell Light™ early endosome (RFP-Rab5a) marker was purchased from Invitrogen and used at 25m.o.i. A mouse monoclonal antibody to Lef-1 (# 17-604) was purchased from Millipore (Billerica, MA).

### Molecular biology

cDNAs encoding wild-type and mutant human SNX27b were generated as described [11]. β-Catenin, tcf-4 expression constructs and TOPFLASH/FOPFLASH luciferase reporter plasmids were kindly provided by Prof. Hans Clevers (Hubrecht institute, Utrecht) [26]. Generation of mutant β-catenin lacking the C-terminal PDZ-binding motif (ΔFDTDL) was performed with Quikchange™ site-directed mutagenesis kit (Stratagene, La Jolla, CA) using the forward primer 5′-CAATCAGCTGGCCTAGTTTGATACTGACCTG-3′ and reverse 5′-CAGGTCAGTATCAAACTAGGCCAGCTGATTG-3′. All cDNA constructs were sequenced to verify the absence of undesired secondary mutations.

### Cell culture and transfection

Mouse primary kidney cortical collecting duct cells (mpkCCD) clone 3 cells were kindly provided by Dr Mark Knepper (NHLBI/NIH). MpkCCD and 293T cells were maintained in high glucose Dulbecco’s Modified Eagle Medium (Invitrogen) supplemented with 10% fetal bovine serum (ATCC) in humidified chambers with 5% CO_2_ at 37°C.

MpkCCD cells were transfected using Lipofectamine LTX reagent including Plus reagent (Invitrogen) using manufacturer’s recommendations. Cells were typically used in biochemical and functional assays 24 to 48 h post-transfection. Cells (293T) stably expressing SNX27 silencing and non-silencing shRNAs were generated using an inducible lentiviral shRNA system (pINDUCER10) and selected with puromycin as described in Valdes et al. [11]. shRNA expression of puromycin resistant clones was induced with 1 µg/ml doxycycline for 72–96 h prior to experimental analysis.

### Protein interaction analysis

The purification of GST and GST–PDZ fusion proteins was performed as described previously [11]. For GST-pulldown experiments, mpkCCD cells were lysed in binding buffer 150 (BB150; 50 mM Tris pH 7.6, 150 mM NaCl, 0.2% CHAPS 3-[(3-cholamidopropyl) dimethylammonio]-1-propanesulfonate), 10 mM EDTA plus Complete™ protease inhibitor cocktail (Roche). Protein concentration was determined using the bicinchoninic acid assay (Pierce Biotechnology, Rockford, IL). GST-pulldowns were typically performed using 0.5–1 mg protein and 10–20 µg fusion protein immobilized on glutathione sepharose beads for 2 h at 4°C. The beads were washed three times in lysis buffer and boiled in sample buffer. For immunoprecipitation experiments, cells were lysed in BB150 containing Complete™ protease inhibitors. Immunoprecipitations were performed using 1–2 mg of protein lysate and 30 µl anti c-myc polyclonal antibody conjugated to agarose beads (Sigma). Immune complexes and proteins bound to GST fusion proteins were analyzed by Western blot and detected using β-catenin or SNX27 polyclonal antibodies at 1:1000 dilutions followed by secondary antibodies conjugated to IRdye780 nm (rabbit) or IRdye680 nm (mouse) (LI-COR, Lincoln NE) at 1:10,000 dilutions. Myc tags were detected by Western blotting using the 9E10 monoclonal antibody at a 1:5000 dilution followed by a secondary antibody conjugated to horseradish peroxidase and detected by enhanced chemiluminescence (Millipore). Experiments to determine the direct interaction of SNX27 with *in vitro* translated β-catenin were performed as described [11].

### Peptide pulldown

Wild-type (PPGDSNQLAWFDTDL) and mutant (PPGDSNQLAWFGGGG) β-catenin C-terminal peptides with an amino-terminal linker sequence (SGSG) were synthesized at the Tufts University Peptide Synthesis core (Boston, MA). A biotin moiety was conjugated to the amino-terminal of each peptide to facilitate interaction with streptavidin. Binding assays were performed as described previously [17].

### Mass spectrometry

To identify novel proteins bound to the PDZ domain of SNX27, samples were electrophoresed on SDS-polyacrylamide gels, and protein bands were visualized by staining with Coomassie blue. The band of interest was excised and digested with trypsin at 1:50 enzyme/substrate ratio. The dried tryptic digest was analyzed on an LTQ-Orbitrap XL (Thermo-Fisher Scientific LLC) interfaced with an Eksigent nano-LC 1D plus system (Eksigent Technologies LLC, Dublin, CA) using CID fragmentation. Samples were loaded onto an Agilent Zorbax 300SB-C18 trap column at a flow rate of 5 µl/min for 10 min, and then separated on a reversed-phase PicoFrit analytical column (New Objective, Woburn, MA) using a 40-min linear gradient of 2–40% acetonitrile in 0.1% formic acid at a flow rate of 300 nl/min. LTQ-Orbitrap XL settings were as follows: spray voltage 1.5 kV; full MS mass range: *m/z* 200 to 2000. The LTQ-Orbitrap XL was operated in a data-dependent mode; i.e*.* MS1 in the ion trap, scan for precursor ions followed by six data-dependent MS2 scans for precursor ions above a threshold ion count of 2000 with collision energy of 35%. The raw file generated from the LTQ-Orbitrap XL was analyzed as described [11].

### Immunofluorescence

MpkCCD cells (3 × 10^5^) were plated on square glass coverslips (484 mm^2^) and transfected as indicated above. Cells were fixed with 4% paraformaldehyde (PFA) for 10 min, permeabilized with 0.1% Triton-X 100 for 5 min, and incubated in blocking buffer (1% bovine serum albumin (BSA) in PBS) for 1 h at 37°C. The fixed coverslips were incubated with β-catenin and EEA1 at 1:200 dilution in blocking buffer overnight at 4°C. Coverslips were washed extensively with PBS and incubated with the indicated secondary antibodies (Alexafluor 488 nm or 597nm (Invitrogen-Molecular Probes) at 1:1000 dilutions) in blocking buffer for 1 h at 37°C. Following further washing with PBS, coverslips were mounted using FluorSave™ (EMD Millipore, Billerica, MA). Wide-field images were collected using a Personal DeltaVision system (Applied Precision Inc, Issaquah, WA, U.S.A.) mounted on an inverted Olympus IX71 microscope with an oil immersion PlanApo N 60×/1.42 objective lens. All images were acquired using a CoolSNAP ES2 CCD camera with 2 × 2 binning and a 512 pixels × 512 pixels imaging field. All images were deconvolved using an iterative constrained method with 10 cycles, medium noise filtering in Applied Precision’s SoftWoRx software package version 4.0.0.

### Immunopurification of the early endosome

The immunopurification of endosomes was performed using an antibody against early endosome marker, EEA1 [27]. Briefly, confluent mpkCCD cells in 6 × 15 cm diameter dishes were removed by treatment with 3 mM EDTA. The cells were centrifuged and the pellet resuspended in 10 mM Hepes buffer, pH 7.2, containing 100 mM KCl, 1 mM EDTA and 25 mM sucrose. The resuspension was passed 15× through a 25-gauge needle and centrifuged twice (3000***g***, 10 min). The supernatant was incubated with monoclonal EEA1 or IgG control antibodies (2 µg per 2 mg lysate, overnight 4°C). The immunoprecipitates were collected using protein G-sepharose beads, and prepared for SDS-PAGE electrophoresis.

### Luciferase reporter assay

To examine the role of SNX27 on the Wnt-dependent transcriptional activity of β-catenin, 293T cells (1.5 × 10^5^) were seeded per well of a 24-well plate and transfected with 150 ng TOPFLASH or FOPFLASH reporter [26] and 20vng β-galactosidase expression plasmid using Fugene HD (Roche Applied Science, Indianapolis, IN). After 8 h, the transfection media was removed and replaced with media supplemented with a ‘pulse’ of Wnt-3a (100 ng/ml) for 16 h, followed by a ‘chase’ in the absence of Wnt for the indicated times, by which time luciferase and β-gal readings were measured. To examine the role of the C-terminal of β-catenin on transcriptional activity, 293T cells (5 × 10^5^) were seeded in 3.5 cm diameter dishes and transfected with 0.67 µg of mCherry-β-catenin (wt or ΔFDTDL), HA-Tcf-4 and either TOPFLASH or FOPFLASH luciferase reporter plasmids using Fugene HD. Luciferase measurements were taken 48 h post-transfection.

## Results

### Identification of β-catenin as a novel SNX27-interacting protein

**Figure 1:**
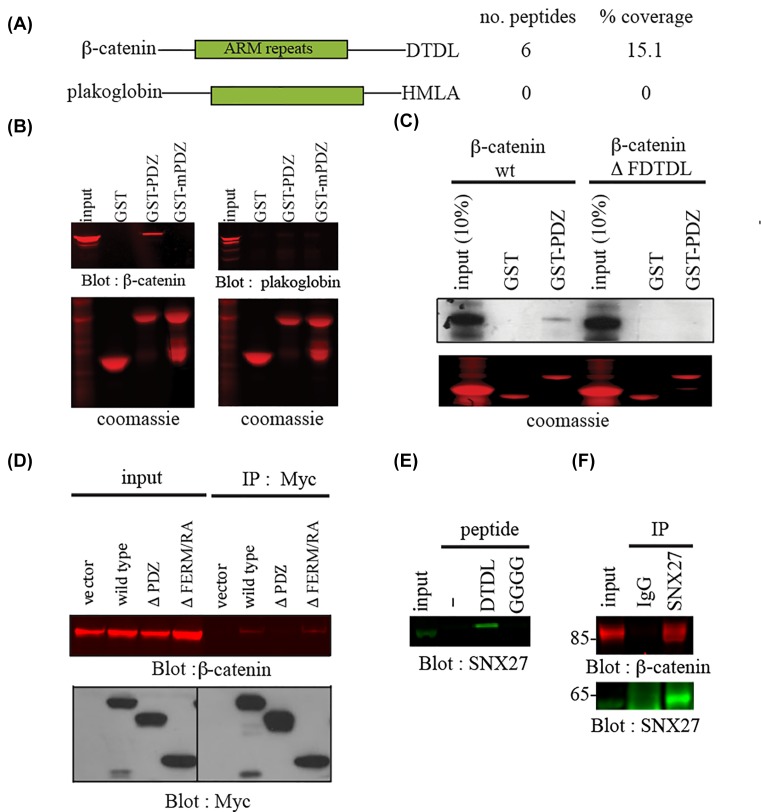
SNX27 binds β-catenin in mpkCCD cells (**A**) About 10 mg of mpkCCD cell lysate was incubated with a GST-fusion protein expressing the PDZ domain of SNX27. Interacting proteins were visualized by staining with Coomassie blue and identified by mass spectrometry as described in ‘Methods’ section. β-Catenin, but not closest relative plakoglobin, was detected in GST-PDZ bound complexes. (**B**) About 500 µg of mpkCCD was incubated with 10 µg GST or GST fusion proteins containing the SNX27 wild-type (GST-PDZ) or mutant (GST-mPDZ) PDZ domain known to abolish carboxy-terminal peptide ligand interactions. After washing, bound β-catenin or plakoglobin was detected by Western blotting. About 50 µg of lysates were loaded as control for Western blotting. Equal GST loading was verified by Coomassie staining. (**C**) About 1 µg of cDNAs of β-catenin wild-type or mutant lacking carboxy terminal PDZ-binding motif (ΔDTDL) were translated in the presence of [^35^S]-methionine. The translated product was incubated with GST or GST-fusion protein encoding the PDZ domain of SNX27 for 1 h at 4°C. The beads were washed, and bound translated product separated by SDS-PAGE. Detection of bound product was revealed by autoradiography. Twenty per cent of the translated product was used as input control. (**D**) MpkCCD cells were transiently transfected with empty Myc-tagged vector or plasmids containing Myc fusion proteins of the indicated SNX27 sequences. After 48 h incubation, the expressed fusion proteins were immunoprecipitated from cell lysates using a Myc antibody, and the immune complexes blotted for β-catenin (top) or Myc (bottom). (**E**) MpkCCD cell extracts were incubated with the indicated wild-type (DTDL) or mutant (GGGG) carboxy-terminal β-catenin peptides conjugated to streptavadin-agarose beads. Bound SNX27 was detected by Western blotting. (**F**) mpkCCD cell lysates (2 mg) were incubated with a SNX27 polyclonal antibody or preimmune serum (5 µl). Immune complexes were collected using protein A-Sepharose. Bound β-catenin was detected by Western blotting. About 50 µg of cell lysate was run as input control.

Using a previously described proteomic approach, we sought to identify novel interacting proteins with the PDZ domain of SNX27 [11]. A GST-SNX27 PDZ fusion protein (GST–PDZ) was incubated with mouse kidney epithelial cell lysates (mpkCCD). Interacting proteins were separated by SDS-PAGE, visualized by Coomassie blue staining and identified by matrix-assisted laser desorption/ionization mass spectrometry (MALDI-MS/MS). At approximately 90 kD, β-catenin was found in GST-PDZ but not control GST-bound complexes (6 peptides, 15.11% coverage) (Figure 1A and Supplementary Table S1). β-Catenin belongs to the armadillo family of proteins characterized by a series of repeat (termed ‘Arm’) sequences in the central domain [28]. A second family member, plakoglobin contains similar repeat sequences but was not found in GST-PDZ bound complexes (Figure 1A.). To validate the specific binding of SNX27 to β-catenin but not plakoglobin, GST-pulldown experiments were performed (Figure 1B). Lysates from mpkCCD cells were incubated with GST alone, GST-PDZ or a mutant PDZ protein where a conserved GΦGΦ (GYGF in SNX27) is mutated to GGGG (mPDZ) as previously described [11]. GST beads were washed and bound β-catenin or plakoglobin identified by Western blotting. Whereas β-catenin did not bind to GST or GST-mPDZ, it was associated with GST-PDZ in both cell lines tested (Figure 1A, left). Plakoglobin did not bind to any GST proteins (Figure 1A, right).

β-Catenin contains a known type I PDZ-binding motif (DTDL), which is absent in plakoglobin (HMLA) (Figure 1A). We hypothesized that these carboxy-terminal residues mediated the direct interaction with SNX27 (Figure 1C). To this end, *in vitro* translated full-length or mutant β-catenin lacking the carboxy-terminal five residues (ΔFDTDL) was incubated with GST alone or GST-PDZ (Figure 1C). Direct binding of β-catenin wild-type to GST-PDZ but not control GST alone was detected (Figure 1C). In contrast, binding of β-catenin ΔFDTDL to both GST-PDZ and GST control beads was not observed (Figure 1C).

To further verify that the PDZ domain on SNX27 was involved in the β-catenin interaction, a series of Myc-tagged SNX27 constructs were expressed in mpkCCD cells and isolated by immunoprecipitation [11]. SNX27-bound β-catenin was identified by Western blotting (Figure 1D). β-Catenin was detected in Myc-SNX27 wild-type and ΔFERM immunoprecipitates, but Myc-mPDZ and Myc-ΔPDZ mutants were defective for β-catenin binding (Figure 1D).

To confirm that the carboxy-terminus of β-catenin mediates the SNX27 interaction, biotinylated β-catenin peptides corresponding to the wild-type (DTDL) or mutant (GGGG) β-catenin carboxy-terminus were synthesized. The peptides were immobilized with streptavidin beads, incubated with mpkCCD cell lysates and the presence of bound SNX27 detected by Western blotting (Figure 1E). Wild-type but not mutant carboxy-terminal β-catenin peptide readily bound SNX27 (Figure 1E). Finally, to determine whether the SNX27–β-catenin interaction occurs at normal physiological levels, endogenous SNX7 was immunoprecipitated from mpkCCD cells at confluence. Endogenous β-catenin was detected in the SNX27 immune complex but not the preimmune control (Figure 1F). Thus, we conclude that SNX27 associates with β-catenin under normal physiological conditions via the PDZ domain of SNX27 and the type I PDZ-binding motif of β-catenin.

### Recruitment of β-catenin to the early endosome by SNX27

**Figure 2:**
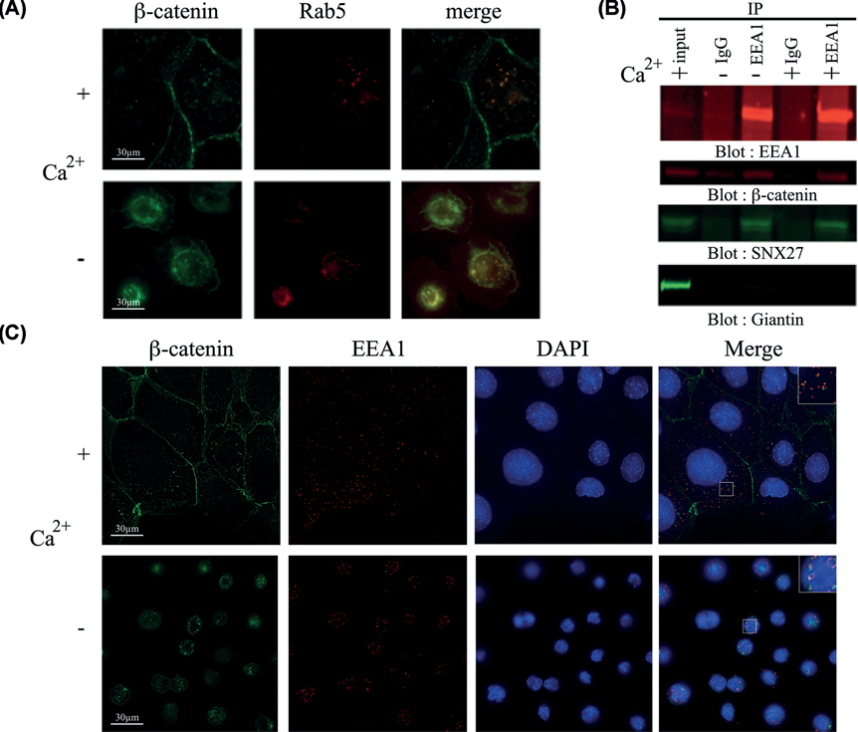
A pool of β-catenin is localized to the early endosome (**A**) MpkCCD cells were infected with RFP–Rab5a (early endosome marker) and grown to confluence. At this point, the cells were depleted of calcium using 5 mM EGTA for 1 h or maintained in normal growth conditions. The cells were fixed and permeabilized and stained with a β-catenin monoclonal antibody and visualized using Alexa-Fluor® anti-mouse 488 nm. (**B**) MpkCCD cells at confluence were depleted of calcium using 5 mM EGTA for 1 h or kept in normal growth conditions. The plasma cell membranes were disrupted and endosomes immunopurified using an antibody against the early endosome marker EEA1. The presence of SNX27, β-catenin or giantin was detected by Western blotting. About 50 µg of cell lysate was run as input control. (**C**) MpkCCD cells at confluence were depleted of calcium using 5 mM EGTA for 1 h or kept in normal growth conditions. The cells were fixed and permeabilized and stained with a β-catenin monoclonal antibody (488 nm) and EEA1 polyclonal antibody (594 nm).

Numerous reports have localized SNX27 to the early endosome in a variety of cell types [11,13,14], while β-catenin has well-characterized roles at the adherens junction and nucleus (reviewed in [29]). To account for the observed SNX27–β-catenin interaction in cells, we proposed that β-catenin might traverse the endosomal network during adherens junction remodeling. To this end, the localization of endogenous β-catenin in mpkCCD cells at confluence and also when cell–cell contacts are ablated by calcium removal was assessed by immunostaining (Figure 2A,C). The early endosome was marked by expression of a RFP–Rab5 fusion protein (Figure 2A) or antibody staining with EEA1 (Figure 2C). As expected, β-catenin was readily detected at sites of cell–cell contact. However, a pool of β-catenin was also detected in cytoplasmic vesicles, which overlapped with the RFP–Rab5 (Figure 2A, top row). A similar vesicular pool of β-catenin was also detected in mpkCCD cells depleted of calcium (Figure 2A, bottom row). Similarly, normal physiological levels of β-catenin and EEA1 were found to colocalize in mpkCCD cells (Figure 2C). To examine the endosomal localization of β-catenin biochemically, intact endosomes from both confluent mpkCCD cells depleted of calcium or maintained under physiological conditions were immunopurified using the early endosome marker EEA1 (Figure 2B). The presence of β-catenin and SNX27 in EEA1 immunoprecipitates was identified by Western blotting. To confirm purity of the endosomal purification, a control blot for giantin, a Golgi-membrane protein marker was also performed. While a small amount of both SNX27 and β-catenin was detected non-specifically in control immunoprecipitates, both proteins were readily detected in EEA1 immunoprecitates under all test conditions (Figure 2B). In contrast, giantin was not detected in endosomal purifications (Figure 2B).

**Figure 3:**
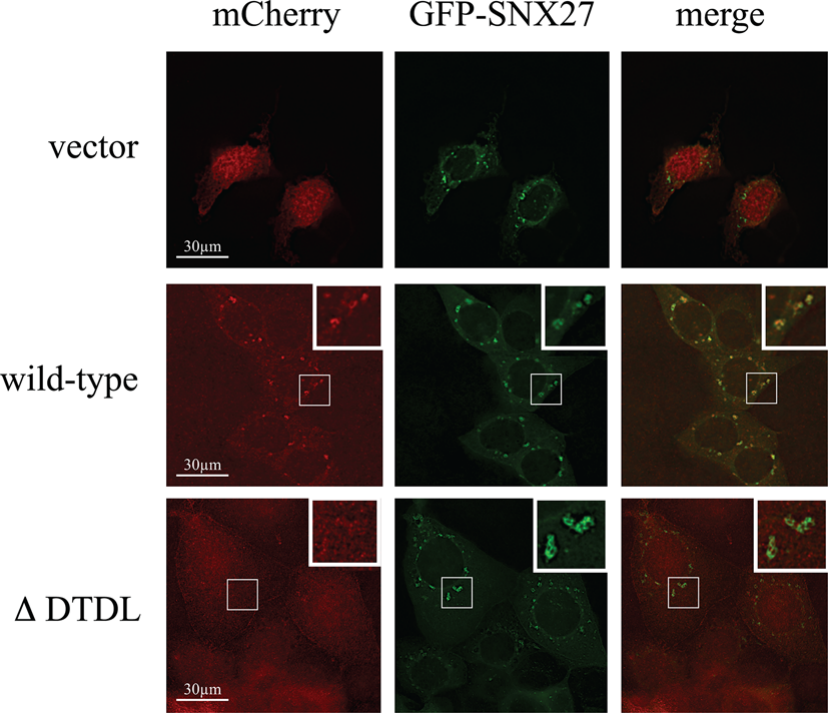
Recruitment of β-catenin to the early endosome is SNX27-dependent mpkCCD cells were contransfected with the indicated GFP-SNX27 or mCherry-β-catenin fusion proteins. After 24 h, the cells were fixed, and permeabilized as described in ‘Methods’ section. β-Catenin and SNX27 expressing cells were identified by mCherry and GFP, respectively.

Having confirmed both the biochemical interaction of SNX27 with β-catenin and the endosomal localization β-catenin in mpkCCD cells, we postulated that SNX27 might be responsible for the recruitment of β-catenin to the early endosome (Figure 3). To test this hypothesis, GFP-tagged SNX27 was coexpressed with a series of mCherry-tagged β-catenin (wild-type, Δ FDTDL or vector control) plasmids in mpkCCD cells (Figure 3). Twenty-four hours post-transfection, the cells were fixed and visualized by GFP or mCherry. The expression of full-length fusion proteins was confirmed by Western blotting (Supplementary Figure S1). As expected, GFP-SNX27 was detected at endosomal sites. In GFP-SNX27 expressing cells, mCherry-β-catenin wild-type protein was detected at both cytoplasmic, with a small amount at the cell periphery. In addition, a pool of β-catenin tightly co-localized with GFP-SNX27 at the early endosome (Figure 3, middle row). In contrast, though mCherry-β-catenin ΔFDTDL was also detected at both the cell periphery and cytoplasm, no overlap with GFP-SNX27 was observed (Figure 3, bottom row). In summary, we have shown evidence of a novel interaction between the PDZ-domain of SNX27 and the type I PDZ binding motif of β-catenin. Furthermore, this interaction is sufficient for the recruitment of β-catenin to the early endosome.

### Cadherin proteins are found in SNX27–β-catenin protein complexes

**Figure 4:**
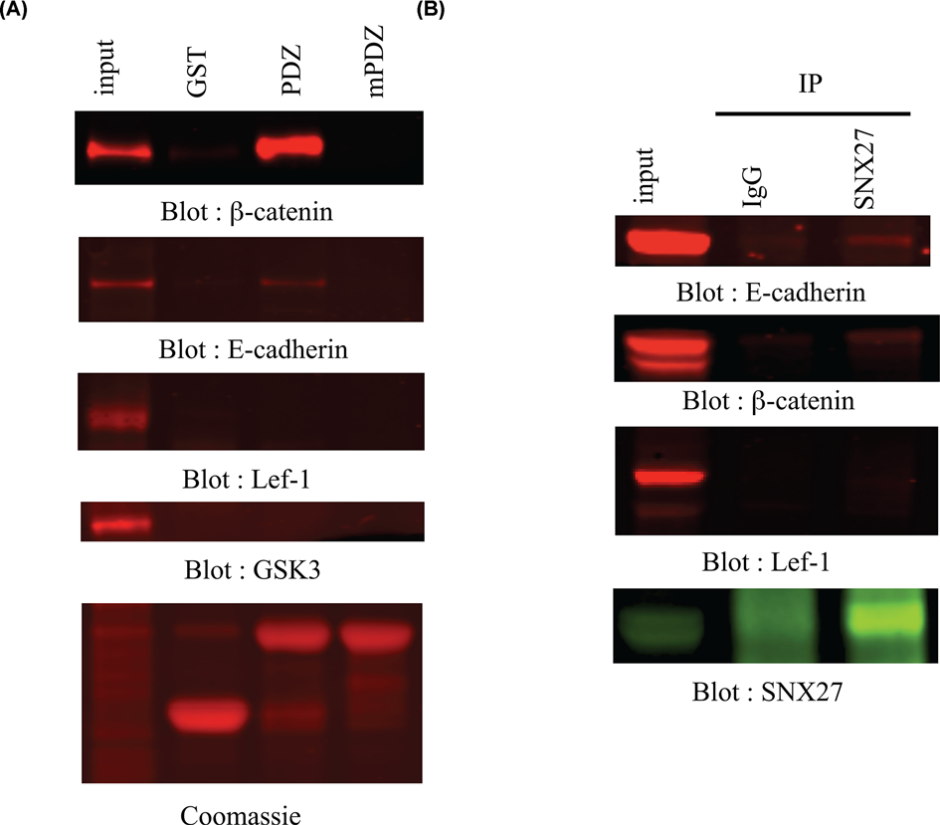
Adherens junction but not Wnt-signaling pathway components are found in SNX27–β-catenin complexes (**A**) About 1 mg mpkCCD were incubated with 20 µg of GST or GST fusion proteins containing the SNX27 wild-type (GST-PDZ) or mutant (GST-mPDZ) PDZ domain known to abolish carboxy-terminal peptide ligand interactions. After washing, bound β-catenin (top row), E-cadherin (second row), Lef-1 (third row) or GSK3 (fourth row) was detected by Western blotting. About 50 µg of lysates were loaded as control for Western blotting. Equal GST loading was verified by Coomassie staining (bottom row). (**B**) MpkCCD cells were lysed at confluence and the cell extracts were incubated with a SNX27 polyclonal antibody or preimmune serum. Immune complexes were collected using protein A-Sepharose. Bound E-cadherin (top row), β-catenin (second row) or Lef-1 (third row) was detected by Western blotting using specific antibodies. Immunoprecipitation of SNX27 was confirmed by Western blotting using a SNX27 polyclonal antibody (bottom row).

We have previously shown a role for SNX27 as a protein scaffold [11]. The binding of SNX27 to the carboxy-terminus of β-catenin prompted the hypothesis that other known-binding partners to β-catenin, such as cadherin, APC, LEF or axin may simultaneously interact with the SNX27–β-catenin complex. To this end, GST-pulldown experiments and co-immunoprecipitation experiments were performed (Figure 4). Lysates from mpkCCD cells were incubated with GST, GST-PDZ or GST-mPDZ and bound β-catenin, E-cadherin, LEF-1 and GSK-3 identified by Western blotting (Figure 4A). As expected GST-PDZ avidly bound β-catenin. In addition, E-cadherin was readily detected in GST-PDZ pulldowns (Figure 4A). In contrast, LEF-1 or GSK3 did not bind to any GST proteins (Figure 4A). In support of the pulldown data, endogenous E-cadherin and β-catenin, but not LEF-1, were precipitated with SNX27 (Figure 4B).

### Effect of SNX27 on Wnt-signaling

The cytoplasmic unbound pool of β-catenin is tightly regulated and maintained at low levels through proteosomal degradation. However, in the presence of Wnt-signaling the pool of cytoplasmic β-catenin is elevated leading to the formation of nuclear transcriptional complexes and elevated transcription of specific Wnt target genes [29,30]. We also postulated whether SNX27 might function to sequester the cytoplasmic pool of β-catenin to influence Wnt-dependent cell signaling.

**Figure 5:**
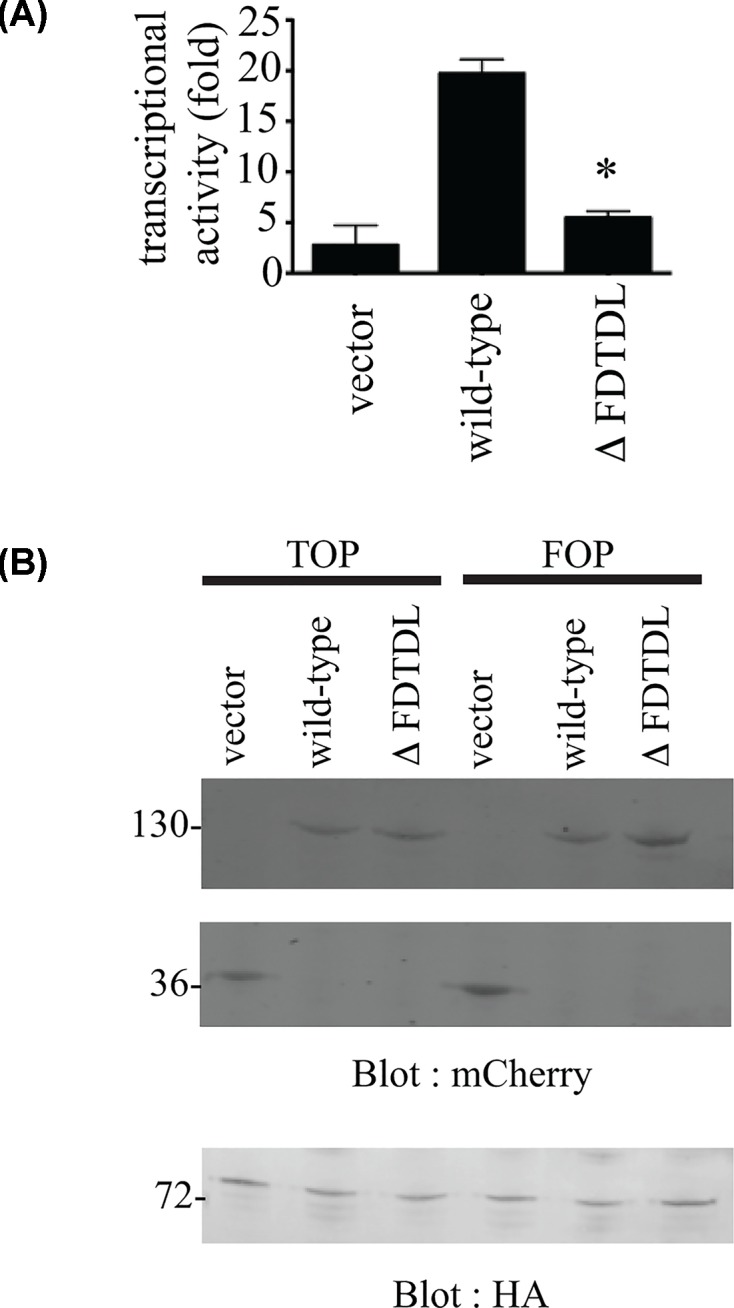
Role of the β-catenin PDZ binding motif to transcriptional activity 293T cells were cotransfected with equal amounts of the indicated mCherry-tagged β-catenin wild-type, mutant (ΔFDTDL) or vector control plasmids along with HA-tagged tcf-4, and either TOPFLASH or FOPFLASH reporters. Total cell lysates were equalized for protein concentration and assayed for luciferase activity 48 h post-transfection (**A**). Expression of β-catenin and tcf-4 was detected using Western blotting using mCherry of HA monoclonal antibodies, respectively (**B**) (*P<0.05).

Using a well-characterized luciferase reporter system, we examined whether the SNX27–β-catenin interaction could modulate Wnt-dependent increase in β-catenin transcriptional activity [26]. First, we examined the importance of the PDZ-binding motif of β-catenin for its function as a transcriptional co-activator. mCherry vector, mCherry tagged β-catenin wild-type and mutant ΔFDTDL were co-expressed with HA-tagged TCF4 and TOPFLASH/ FOPFLASH luciferase reporter plasmids (Figure 5). As expected, an approximately 20-fold increase in transcriptional activity was observed in cells expressing wild-type β-catenin (Figure 5A). In contrast, reporter activity in cells expressing β-catenin ΔFDTDL was diminished to control levels (*P* = 0.0003, *n* = 3). Equal protein expression was confirmed by Western blotting for the mCherry and HA tags for β-catenin and TCF, respectively (Figure 5B). These results implied that a protein–protein interaction to the COOH-terminal of β-catenin was important for the transcriptional activity of β-catenin.

**Figure 6:**
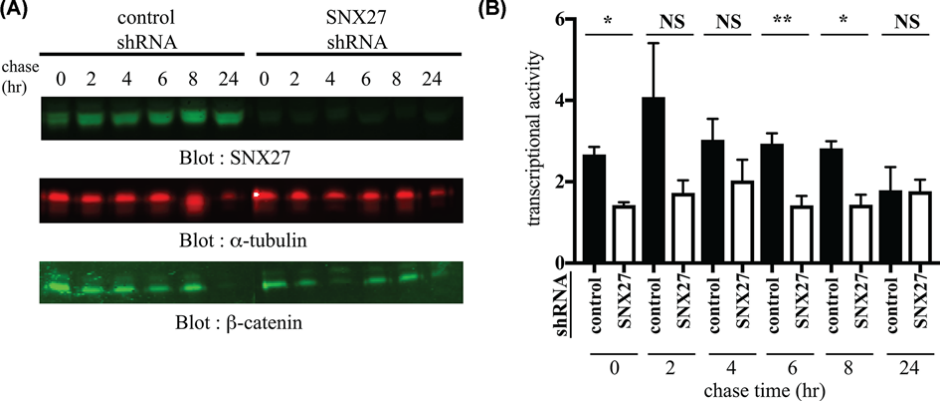
The effect of SNX27 on the transcriptional activity of β-catenin 293T cells were infected with lentiviral SNX27 or control shRNA and selected with puromycin. shRNA expression was induced with 1 µg/ml doxycycline for 48 h. At this time, the cells were transfected with TOPFLASH or FOPFLASH luciferase reporter plasmids. Eight hours post-transfection, the cells were pulsed with 100 ng/ml Wnt3A for 16 h then chased in the absence of Wnt3A for the indicated times. Cell lysates were assayed for SNX27 (top), α-tubulin (middle) or β-catenin (bottom) expression by Western blotting (**A**) and luciferase activity (**B**).

If SNX27 binding was important for β-catenin transcriptional activity, then the above results could be recapitulated in 293T cells lacking SNX27. We transfected TOPFLASH or control FOPFLASH reporter into 293T cells lacking SNX27 and ‘pulsed’ the transfected cells with 100 ng/ml Wnt3a for 16 h, followed by a ‘chase’, where the Wnt stimulus was removed for the indicated times, at which point reporter activity was measured. As expected, Wnt3a treatment led to elevated β-catenin protein levels in both control and SNX27shRNA expressing cells at ‘chase’ time 0 (data not shown) which, in the absence of Wnt3A diminished over time at a similar rate in both control and SNX27-depleted cells (Figure 6A).

As expected, 293T cells treated with Wnt3A led to an increase in β-catenin-TCF/LEF transcriptional activity (Figure 6B). However, in cells lacking SNX27, the Wnt-3a dependent increase in β-catenin-TCF/LEF transcriptional activity was consistently lower than in control cells in all “chase’ times from baseline to 8 h. After 24 h of Wnt3A removal, the levels of both β-catenin protein and transcriptional activity had returned to baseline in both control and SNX27 knockdown cells (Figure 6A,B).

## Discussion

Proteins of the sorting nexin (SNX) superfamily are characterized by the presence of a phox-homology (PX) domain and associate with phosphatidylinositol-3-monophosphate (PtdIns3P) rich regions of the endosomal system. SNX27 is the only sorting nexin that contains a PDZ domain. Here, we identified a novel interaction between SNX27 and β-catenin. The PDZ domain of SNX27 and C-terminal residues of β-catenin mediated the interaction.

While β-catenin is mainly regulated by multiple protein–protein interactions with the central ‘Arm’ repeat motifs [29,31–33], an unstructured carboxy-terminal domain is involved in the recruitment of chromatin-dependent factors [31]. Furthermore, the last four amino acids of β-catenin (DTDL) have been shown to bind the proteins Scribble, Tax-interacting protein 1, LIN-7 and members of the Na^+^/H^+^ exchanger regulatory factor NHERF and membrane-associated guanylate kinase protein families suggesting that this mechanism of protein–protein interaction may regulate the distribution and function of β-catenin at different sites of the cell and in multiple cell types [34–39].

Numerous studies have localized SNX27 to the early endosome, yet β-catenin predominantly is found at the nucleus and at sites of cell–cell contact [11,13,14,33,40]. To reconcile this difference, it is plausible that a pool of β-catenin might use the endocytic system to traffic between cell–cell contact and nuclear sites. The endocytosis of cadherin proteins in a clathrin-dependent manner and delivery to the endocytic network has long been known. Plus, endosomal β-catenin has been observed [24,41–43]. In the present study, we also noted a pool of β-catenin in the early endosome of mpkCCD cells, both in conditions of cell confluence or when cell-cell contacts are lost. Furthermore, by overexpression of fluorescently tagged proteins, we demonstrated the importance of SNX27 binding for the recruitment of β-catenin to the early endosome.

The remaining part of the present study considered the functional consequence of SNX27 binding on β-catenin function. Given the restricted expression of SNX27 to the endosome, we proposed that SNX27 might function to sequester β-catenin alone, or in complex with known binding partners to endosomal sites to regulate the trafficking of β-catenin to the nucleus to modulate transcription or to the adherens junction to modulate cell–cell contacts.

By biochemical methods, we detected E-cadherin, but not LEF-1 or GSK-3 in SNX27–β-catenin complexes in confluent mpkCCD cells. This suggested that SNX27 might specifically regulate trafficking of β-catenin and E-cadherin to or from the adherens junction, rather than participate in Wnt signaling. However, the cellular localization of both β-catenin and E-cadherin in mpkCCD cells lacking SNX27 was unchanged in cells at confluence. Furthermore, the rate of recycling of endogenous β-catenin and E-cadherin to the adherens junction in cells lacking SNX27 post-calcium switch was also unaltered (data not shown). However, we may be overlooking the fact that the intracellular trafficking of β-catenin may be highly dynamic. Hence, we attempted FRAP (fluorescence recovery after photobleaching) experiments of N-terminal tagged mCherry-β-catenin under live cell conditions in mpkCCD cells lacking SNX27 [17]. However, these experiments were unsuccessful as we found that an overexpressed an N-terminal tagged mCherry–β-catenin was only minimally observed at sites of cell contact. It is interesting to note that expression of a C-terminal GFP-tagged β-catenin was found to localize to both nuclear and endosomal structures [43].

Numerous mechanisms have been proposed by which the cytoplasmic pool of β-catenin is regulated (summarized in Li et al.) [44]. There is also precedence that the endosomal network may regulate β-catenin cytoplasmic accumulation and gene expression [45–47].

We postulated that this small SNX27-bound endosomal pool of β-catenin may regulate cytoplasmic levels and downstream transcriptional activity of β-catenin.

To address this hypothesis, we first compared the transcriptional activity of full-length β-catenin with a SNX27-binding defective mutant (ΔFDTDL). The protein stability and relative nuclear/cytoplasmic distribution of both mutant and wild-type proteins were found to be similar (data not shown), which is in agreement with previous data from the Gottardi laboratory [37,48]. However, the transcriptional activity of mutant β-catenin was suppressed to control levels. Similarly, Wnt-mediated stimulation of β-catenin transcriptional activity is recapitulated in cells lacking SNX27.

There is a report of one other PDZ binding motif-containing protein that may regulate the function of β-catenin as a transcriptional co-activator. As previously reported by Sun et al*.* one of the receptors that binds Wnt, Fzd7 is a SNX27–PDZ interacting protein [49]. It is therefore plausible that our reported defective β-catenin transcriptional activation in cells expressing SNX27-ΔFDTDL may be additional due to defective Fzd7 signaling.

In summary, we present SNX27 as a novel β-catenin interacting protein which is an important player in the complex and fascinating role of β-catenin in normal and disease pathology. The observations that the SNX27-β-catenin interaction may induce a decrease in transcriptional activity suggests that inactivation of this protein-protein interaction might be beneficial in the treatment of β-catenin-dependent tumors.

## Supplementary Material

Supplementary Figure S1

Supplementary Table S1

## Abbreviations

PFA: paraformaldehyde
PX: phox homology
SNX27: sorting nexin 27
SNX: sorting nexin
