# Supplementary material for: Characterization of the interaction between β-catenin and sorting nexin 27: contribution of the type I PDZ-binding motif to Wnt signaling
Source: Biosci Rep. 2019 Nov 13;39(11):BSR20191692. doi: 10.1042/BSR20191692 (PMC6851508; doi:10.1042/BSR20191692)
Supplement: Supplementary Figure S1 [file BSR-2019-1692_supp.pdf]

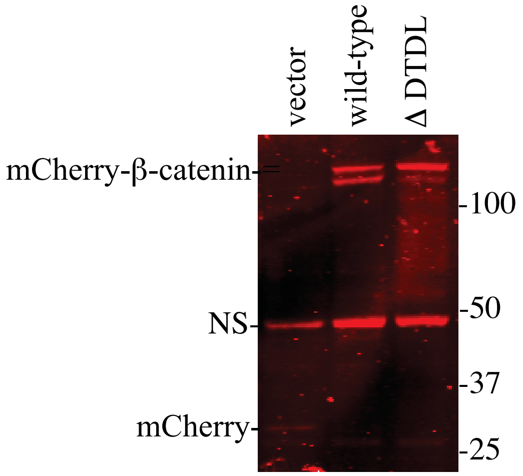

Supplementary figure 1

Expression of mCherry fusion proteins in mpkCCD cells.

The indicated mCherry fusion plasmids were transfected in mpkCCD cells. 24hr post-transfection the cells were lysed. Expression of fusion protein detected using western blotting by mCherry specific antibodies.
